# Fit for the Future: An Assessment of the Sustainability Parameters of Liquid Dairy Product Packaging in the DACH Region and the Implications of Upcoming Regulatory Changes

**DOI:** 10.3390/foods14020195

**Published:** 2025-01-09

**Authors:** Michelle Klein, Charlotte Neumair, Mattia Primoceri, Manfred Tacker, Silvia Apprich

**Affiliations:** 1 Section Packaging Technology and Natural Resource Management, University of Applied Sciences Vienna, Favoritenstrasse 226, 1100 Vienna, Austria; silvia.apprich@fh-campuswien.ac.at; 2 Circular Analytics TK GmbH, Canovagasse 7/1/14, 1010 Vienna, Austriam.primoceri@gmail.com (M.P.); manfred.tacker@circularanalytics.com (M.T.)

**Keywords:** dairy, packaging, sustainability, circularity, milk, LCA, recyclability, packaging efficiency, recyclate, assessment

## Abstract

The European Union aims for climate neutrality by 2050 and has proposed the Packaging and Packing Waste Regulation (PPWR) to promote a circular economy, focusing on reducing packaging waste. In this context, a comprehensive sustainability assessment for liquid dairy product packaging, including beverage cartons, bottles and to-go cups, in the DACH region (Germany, Austria and Switzerland) was conducted. The aim was to consider various ecological aspects of environmental impacts and circularity. As the aspect of recyclability is a core aspect in the PPWR, the calculation was of central interest in this project. Here, major differences in the waste management infrastructure between countries could be identified. The majority of assessed packaging falls below the PPWR’s 70% recyclability requirement, with Switzerland showing even lower recyclability due to poor packaging collection and recycling infrastructure. Significant discrepancies in packaging efficiency exist, indicating unnecessary resource consumption, especially in the case of to-go cups. Additionally, the carbon footprint of packaging materials can vary up to ten times within certain product categories, negatively impacting the environment. Good results were identified for the use of certified renewable resources. Overall, the results of the assessment demonstrate several areas for improvement in light of forthcoming regulatory requirements, which must be met in Germany and Austria.

## 1. Introduction

The concepts of sustainability, the circular economy and food systems are of central importance in social and political discussions. A food system encompasses the entire process of food production, processing, distribution, consumption and disposal. A sustainable food system must be economically viable, socially beneficial and have a low environmental impact. This ensures the current food security of the population and guarantees future food availability [1].

The packaging of food plays a pivotal role in the global food distribution system with regard to sustainability. In the context of globalization and evolving consumer demand, the implementation of a circular economy in food packaging processes has become a matter of urgent concern [2].

Packaging serves to inform and attract consumers [3]. The primary function of food packaging, nonetheless, is to protect food [4,5,6,7]. Effective packaging reduces food waste and thereby the associated greenhouse gas (GHG) emissions, particularly for animal products. Packaging itself typically contributes only a small fraction of the GHG emissions of the packaged product [8], representing approximately 4% of the CO_2_ equivalents for milk packaging [9]. In contrast, dairy production in Germany accounts for 24% of the carbon footprint of all packaged foods [10]. Since 93% of all food is packaged, dairy products significantly impact the environment, making the dairy industry crucial for environmental considerations [9,10]. Consequently, the dairy industry represents a significant contributor to the environmental impact of food.

Materials used for food packaging must be affordable, easily processable and provide food protection—often by offering excellent barrier properties against moisture, oxygen and carbon dioxide. Among the materials that meet these criteria, plastics are often the preferred choice for food packaging [11]; however the substitution of conventional packaging materials with more sustainable alternatives, such as microbial biodegradable polymers from agro-food waste residues, could result in a notable reduction in the carbon footprint [12].

In Europe, there is a significant discrepancy between the recycling of packaging and the amount of packaging waste generated within the Member States. There is a clear opportunity to enhance waste management and the recycling of food packaging. The European Circular Economy Plan, adopted by the European Union, mandates more rigorous recycling standards and reduced food waste. The objective of making Europe climate-neutral by 2050 was defined in the ‘European Green Deal’ [13]. The European Union has proposed a legislative framework for a Packaging and Packaging Waste Regulation [14]. This would enforce strict measures to avoid generating packaging waste and promote the reuse and recycling of packaging materials through intelligent design [14]. This also enforces the need to investigate the influence of food packaging design on sustainability and circularity [15,16]

### 1.1. Dairy Products and Requirements for Dairy Packaging

The dairy industry represents the most significant agricultural sector in German-speaking countries. In Germany (19%), Austria (18%) and Switzerland (20%), milk production accounts for approximately one fifth of their respective agricultural sectors, with a produced volume of 33.1 million tons of milk produced in 2020 in Germany, 3.38 million tons in Switzerland in 2019 and 3.9 million tons in Austria in 2022 [17,18,19]. Given the significance of the dairy sector in the DACH region (Germany, Austria and Switzerland) and the considerable volume of dairy products that are packaged, it is imperative to identify optimal packaging solutions for drinking milk and dairy products that align with the principles of the circular economy and the forthcoming Packaging and Packaging Waste Regulation.

Milk, rich in proteins, lactose and fat, has an almost neutral pH, creating an ideal environment for microorganisms that can affect its quality and safety, potentially leading to contamination and alterations in taste. The pasteurization or sterilization of milk prior to packaging eliminates microorganisms. Ultra-high temperature (UHT) milk is subjected to a sterilization process and subsequently stored at room temperature, whereas pasteurized milk is refrigerated. It is of vital importance to ensure that milk is stored in accordance with the requisite conditions in order to maintain its quality and safety. Furthermore, the packaging must protect against oxygen, light and undesirable odors and flavors from the environment, whilst ensuring no interaction with the milk, in order to avoid the risk of the migration of the packaging components [15,20,21].

Transparent glass and plastic packaging materials fail to provide sufficient protection against harmful wavelengths. The utilization of colored packaging materials or beverage cartons offers an enhanced light barrier. Plastic bottles manufactured from HDPE can be rendered more impermeable to wavelengths below 390 nm through the addition of titanium dioxide [20,21]. The presence of oxygen, particularly in combination with light, causes fat oxidation and leads to the development of undesirable flavors in milk. Glass itself is completely impermeable to oxygen and therefore protects the product from additional oxygen ingress. Non-aseptic beverage cartons consisting of PE-coated cartons give sufficient protection for fresh milk with a short shelf life. For products with a longer shelf life, such as UHT milk, beverage cartons require an additional barrier (e.g., aluminum foil) as an intermediate layer to provide additional protection against oxygen and light [20,21].

As milk is particularly susceptible to the absorption of foreign flavors or tastes, it is crucial to select packaging materials with low aroma permeability. Glass, metals and polyester are highly impermeable to flavors, while PE-coated paper or cardboard is highly permeable. Here an additional barrier, such as aluminum foil, has proven beneficial for proper protection [20,21].

### 1.2. Holistic Sustainability of Packaging

In order to ensure the sustainability of plastics, it is vital to prevent their disposal into the environment. Furthermore, it is of the utmost importance to appreciate the value of plastics and to design packaging in a way that allows for the highest degree of circularity [22,23].

An holistic sustainability assessment is designed to evaluate all relevant aspects of the sustainability of packaging, including country-specific collection and recycling systems. This approach is essential for sustainable product development, as it allows for the consideration of potential conflicting objectives. This model is based on the three pillars of product protection, circularity and the environment, as outlined in the Circular Packaging Design Guideline [4,24].

According to the Proposal for the Packaging and Packaging Waste Regulation, recyclable packaging is specifically designed for recycling, can be efficiently and effectively collected separately and sorted into defined waste streams and can be recycled at large scale into secondary raw material of a quality such that primary raw material can be substituted [14].

The Efficient Consumer Response (ECR) Guideline as well as the Circular Packaging Design Guideline define direct and indirect environmental impacts [4,24]. Direct impacts, related to the production and disposal of packaging, can be assessed through a life cycle assessment. Indirect impacts can stem from product losses, such as premature spoilage or poor emptying due to poor packaging design and are not included in the life cycle assessment, as they are subject to scenarios. Non-quantifiable factors include the use of certified materials, which has a positive impact on the sustainability assessment, and the ‘littering potential’ (the potential for packaging or packaging components to end up in the environment instead of being disposed of properly) [24].

### 1.3. Packaging Design for Recycling

The efficiency of recycling is contingent upon the design of the packaging. In order for packaging to be recycled, it must undergo collection, sorting and recycling processes. Designing for recycling is of essential importance for sustainable packaging and is a requirement of the current proposal for the PPWR [14]. Therefore, packaging should be designed with reusable and renewable properties in order to minimize its environmental impact [25,26].

This study was conducted to evaluate and compare the sustainability of currently used packaging formats for liquid dairy products in the DACH region based on selected parameters. Further, the potential for the improvement of the available packaging on the market regarding the aforementioned PPWR will be discussed.

## 2. Materials and Methods

Prior to the start of the project, relevant product categories for assessment were defined and packaging formats available in selected supermarkets in Germany, Austria and Switzerland identified.

### 2.1. Market Screening

Market screening was conducted in two selected full-assortment supermarkets (SPAR and Billa) and one discounter (Hofer) in Austria. All dairy products from the relevant product categories available online and offline were listed, and detailed information concerning product types in combination with packaging specificities was analyzed for each product category. The supermarkets SPAR and Billa have a market share of 34.6% and 33.3%, respectively, in Austria; the share of HOFER is approximately 20%. Therefore, an overall market share of 88% has been analyzed [27]. The market research was extended to Germany and Switzerland for selected product categories in order to assess country-specific differences in market offerings, by analyzing the available products and packaging in store.

### 2.2. Materials

The packaging samples assessed in this benchmarking project were provided from dairies or retailers in Germany, Austria and Switzerland. Further samples were procured with the objective of encompassing a diverse array of packaging alternatives for each product category. Overall, 76 samples were evaluated within the framework of the sustainability assessment.

#### 2.2.1. Product Categories

According to Codex Chapter B32 of the Austrian Food Code [28], different types of milk products can be distinguished.

Fresh and ESL (extended shelf-life) milk were assessed together and considered separately from long-life milk, given that aseptic filling with a correspondingly longer shelf life is intended for UHT milk [29].

Overall, 76 products were analyzed. Those can be subdivided into the mentioned product categories as follows:

- Milk Products: (31)
  ○ Fresh and ESL milk (17)
    ■ ESL milk (9);
    ■ Whole milk (4);
    ■ Skim milk (4).
  ○ UHT milk (14)
- Mixed Milk Products: (45)
  ○ Fermented products (20)
    ■ Butter milk (6);
    ■ Yoghurt drink (9);
    ■ Whey drink (5).
  ○ Non-fermented products (25)
    ■ Coffee drink (9);
    ■ Chocolate drink and cocoa (12);
    ■ Protein drink (4).

The category of mixed milk products encompasses all mixed milk products as defined in Codex Chapter B32/Milk and milk products/subcategories 5.1.2 and 5.1.3, in addition to all fermented milk products falling under subcategory 6.2 of the Austrian Food Code [28]. The product category of ‘drinking yoghurt and yoghurt milk’ includes mixed milk beverages whose names include the term ‘yoghurt’. Mixed fruit milk drinks were combined with cocoa, chocolate and vanilla milk. Mixed milk drinks with increased protein content are defined as products with a protein content of at least 20% of the total calorific value of the food, in accordance with Regulation (EC) No 1924/2006 of the European Parliament and of the Council of 20 December 2006 on nutrition and health claims made on foods [30].

In order to evaluate milk-based mixed drinks, it was necessary to distinguish between those products made from non-fermented milk and those made from fermented milk. This categorization is based on subcategories 5.1.2. and 5.1.3 of Codex Chapter B32/Milk and dairy products from the Austrian Food Code. While the Food Codex (subcategories 7.2 and 7.3) treats whey separately, for the purposes of this study it has been categorized as a mixed dairy product made from fermented milk [28].

#### 2.2.2. Packaging Types

As with the products analyzed in this study, the packaging formats available on the market show a high diversity. In the case of liquid dairy products, these were primarily composite beverage cartons of various designs, as well as bottles made of PET, HDPE or PS (Table 1). An LDPE pouch with a 35% chalk content was also included in this segment. Mixed milk drinks were available in cans made of aluminum or paper composites, as well as to-go cups of various designs and materials. Reusable glass systems were not included in this study due to the high variability in the environmental impact caused by transport routes and circulation figures, as well as the resulting differences in the streamlined LCA.

### 2.3. Methods

The data for material composition and packaging measurements were obtained either by an analysis of packaging specifications submitted by the participants of the project, or they were evaluated by measurements in the laboratory using calipers, scales and an FTIR spectrometer (Perkin Spektrum UATR L1600300 Spektrum TWO LiTa, Llantrisant, UK) and attenuated total reflectance (ATR) if necessary.

**Figure 1:**
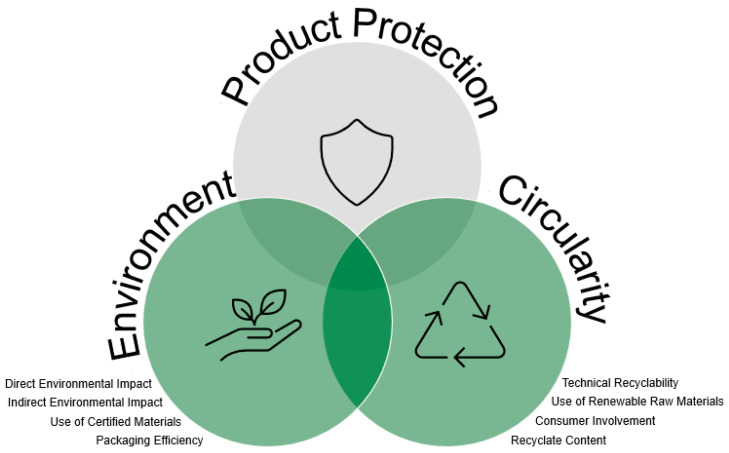
Overview of assessed categories for environment and circularity according to the holistic sustainability assessment for packaging method (adapted version based on [4]).

The selection of the relevant parameters to assess the sustainability of dairy product packaging was based on the model for holistic sustainability assessment which is built on three pillars: product protection, environment and circularity [4]. Eight parameters for circularity and environment were selected, as shown in Figure 1.

#### 2.3.1. Environment: Direct Environmental Impact

Life cycle assessments have been demonstrated to be an effective tool for assessing environmental impacts. The European Commission outlines 16 impact categories for the preparation of life cycle assessments in the so-called product environmental footprint (PEF). The PEF is based on the life cycle assessment (LCA) and measures the environmental impact potential of products. Among the various environmental impact categories that can be quantified through life cycle assessments, the carbon footprint is the most well known. This allows for the assessment of a product’s greenhouse gas emissions, which are subsequently linked to global warming [31,32]. Other impact categories such as acidification or eutrophication are not included in the assessment.

In order to ascertain the direct environmental impact of the packaging, a streamlined life cycle assessment was conducted. All 16 impact categories according to the European PEF were calculated, but only the greenhouse gas emissions are tabled in this study. The LCA is based on the ISO 14040 and ISO 14044 standards [33,34], as well as the European Commission’s regulations for product environmental footprints [31]. For the calculation, information on the material composition and measurements of the packaging are entered into the Packaging Cockpit software, Version 2.0.0 (https://packaging-cockpit.com/en (accessed on 30 September 2024) [35]. The impact category of climate change was considered, and results are expressed in [kg ${\mathrm{CO}}_{2}$ eq].

#### 2.3.2. Environment: Indirect Environmental Impact

The indirect environmental impact was quantified by measuring food residues following the application of newly developed, standardized methods for measuring technical emptiability. In this method, the packaging is emptied according to described consumer handling instructions and based on the further development of prior published emptiability methods. The detailed procedures for each packaging type and the results were published in [36].

#### 2.3.3. Environment: Use of Certified Materials

The utilization of certified materials was evaluated through a qualitative approach. The two selection options were “yes” and “no”. If the packaging incorporated FSC-certified fiber-based materials, the “yes” option was selected. Conversely, if no fiber-based materials were employed or the fibers were not substantiated to be from FSC-certified materials, the “no” option was selected.

#### 2.3.4. Environment: Packaging Efficiency

The packaging efficiency is defined as the ratio of packaging weight to product weight and packaging weight. In the case of filled samples, the actual product weight was included in the calculation. Conversely, in the case of empty packaging samples, the labeled quantity of filling good was included in the formula instead of the weight of the filling good. This method was first applied by [37] as the packaging to product ratio.

Packaging Efficiency=(Weight Packaging/Weight Packaging+Weight Product) × 100

#### 2.3.5. Circularity: Technical Recyclability

Technically recyclable packaging has to fulfill a number of criteria. Firstly, there must be a collection and sorting structure for the materials in their respective countries. Secondly, it must be possible to assign items of packaging to a defined material stream according to the state of the art in their respective countries. Once the packaging has been sorted, it can then be utilized through a corresponding recycling process. The recyclate must be of such a quality that it can be used as a substitute for virgin material with a pricing that has market potential [4]

The technical recyclability of the packaging was calculated for Germany, Austria and Switzerland using the software Packaging Cockpit, Version 2.0.0 (https://packaging-cockpit.com/en, accessed on 30 September 2024) [35]. For this, the detailed packaging information was entered into the software based on the packaging specifications provided with the packaging samples

The data set comprised information pertaining to the filling, including product cate-gory, quantity or volume, country of assembly and distribution, packaging dimensions, the main packaging body type and the types of packaging aids.

In order to complete the assessment, the following information is required for the main body:

- Printing coverage in %;
- Flexible or rigid component;
- Presence of an NIR barrier;
- Information on material layers (each layer needs to be entered, and a detection layer needs to be selected):
  ○ Material;
  ○ Material manifestation;
  ○ Manufacturing type;
  ○ Content of recyclate in %;
  ○ Color;
  ○ Mass in g;
  ○ Material density.

In case the packaging contains a closure, the following data are necessary:

- Type of closure;
- Printing coverage in %;
- Flexible or rigid component;
- Presence of an NIR barrier;
- Dimensions;
- Irreversible removal through consumption/usage;
- Removal for disposal by average consumer;
- Information on material layers (the same information as for the main body is relevant).

If the packaging has decorative elements, additional information is needed:

- Type of decoration;
- Printing coverage in %;
- Covered surface area of main body in %;
- Presence of an NIR barrier;
- Dimensions;
- Irreversible removal through consumption/usage;
- Removal for disposal by average consumer;
- Adhesion to main body;
- Information on material layers (the same information as for the main body is relevant)

Information on the material layer was obtained either from the provided packaging specification or by identification through FTIR-ATR. Dimensions and weights were measured using scales (Ohaus Pioneer Precision, Model PX6202, Nänikon, Switzerland) and calipers, and estimations were made of the print coverage and covered surface area for bottles with sleeves. The assessment method for technical recyclability was based on the current Circular Packaging Design Guideline published by the University of Applied Sciences Campus Vienna [4].

#### 2.3.6. Circularity: Use of Recyclate

Recyclate content is calculated as the weight percentage of post-consumer recyclate of the whole packaging. The use of recycled materials in the production of packaging items reduces the need for new raw materials, thereby contributing to a circular economy [4]. This indicator is equal to the recycled content indicator described by [37].

#### 2.3.7. Circularity: Use of Renewable Resources

The utilization of renewable raw materials is of significant consequence within the context of the circular economy, as it reduces the necessity for non-renewable resources. The results show the proportion of renewable materials as a percentage by weight in relation to the total weight of the packaging, as formerly described by [37].

#### 2.3.8. Circularity: Consumer Involvement

The consumer involvement criterion provides information on the extent to which end consumers are required to perform active separation prior to disposal in order to enable high-quality recycling. If separation by consumers is required, this should be indicated on the packaging, and mechanical separation and subsequent separate disposal should be facilitated, e.g., by perforations. It should be noted that the German minimum standard only permits the separate assessment of packaging components if this is absolutely necessary for the use and consumption of the product [38].

The categories are as follows:

1. Separation action is required from consumers before disposal of the packaging AND sufficiently labeled separation description;
2. Separation by consumers is required before disposal of the packaging AND insufficiently labeled separation description;
3. No separation step by consumers is required before disposal of the packaging.

## 3. Results

The results of the market screening and the assessment of the different parameters for different product categories are presented below. Detailed results can be found in the Supporting Information.

### 3.1. Market Screening

In order to assess the available packaging options on the market, a thorough market screening was conducted (Table 2). This approach enabled the incorporation of a diverse range of packaging types into the sampling. To avoid the inclusion of duplicates, it was necessary to ensure that products were not included more than once in the sampling.

For Austria, the products of the online shops of BILLA, SPAR and HOFER were listed and analyzed regarding the prevalent packaging type. Bottles made of HDPE and PET are denoted as plastic bottles.

### 3.2. Technical Recyclability

The technical recyclability and direct environmental impact were assessed for all product categories separately and for the three countries Germany, Austria and Switzerland.

The most prevalent types of packaging utilized in the dairy beverage sector are composite beverage cartons and PET and HDPE bottles. In the case of fresh and ESL milk, most of the samples were beverage cartons, with the exception of one pouch and two PET bottles. The range of results for all three countries falls between 60.55% and 81.66% in terms of recyclability, due to different waste management systems and different waste streams, as well as slight differences in the material composition and its thresholds. One exception is a PET bottle, which achieved a recyclability score of 99.97%, with minor deductions due to the use of dark printing ink on the sleeve. One illustrative example of the discrepancies in waste management infrastructure between the three countries is the pouch containing skim milk, which exhibited a 99.94% recyclability in Germany, a 98.46% recyclability in Austria and an 0% recyclability in Switzerland, due to the absence of a recycling stream.

In the category for UHT milk, the dispersion of results is similarly narrow, as 13 of the 14 samples are beverage cartons. The range for recyclability lies between 61.65% and 79.80% in all three countries. One outlier is an opaque HDPE bottle with a recyclability of below 1%. As the bottle is made of HDPE, it enters the recycling stream for PE. Thereby, the paper label is considered as contaminating the waste stream, as it has been declared unremovable due to the use of adhesives. Another contributing factor for non-recyclability is the opaqueness of the bottle. The only recyclable component is the aluminum lid of the bottle, which contributes only to 0.8% of the total packaging weight.

**Figure 2:**
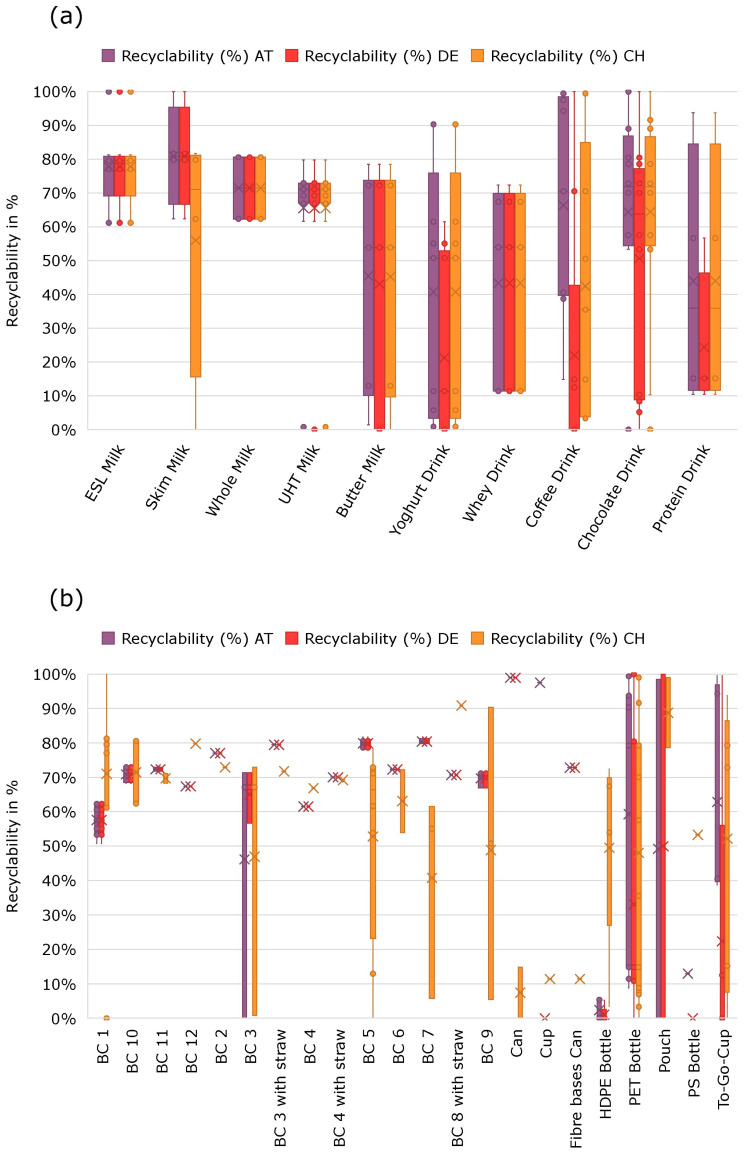
Technical recyclability of product packaging for liquid dairy products for the countries Austria (AT), Germany (DE) and Switzerland (CH) (**a**) in the different assessed categories and sub- groups and (**b**) by comparing packaging types.

Germany, Austria and Switzerland exhibit notable differences in the recyclability of packaging for mixed milk drinks (Figure 2) that are attributable to the variety of packaging employed for mixed milk drinks, which encompasses a range of materials, including to-go cups, aluminum or fiber-based cans, plastic bottles and composite cartons. The DACH region displays considerable disparities in its recycling infrastructure, particularly with regard to to-go cups. The packaging format with the highest recyclability percentage in all three countries was PET bottles with a PET sleeve and an HDPE cap, as well as an aluminum can, with a recyclability of over 99%. The least recyclable packaging was an HDPE bottle with a PS sleeve, an additional paper label and an HDPE cap, with a recyclability of 0% in Austria and Switzerland and a value of 5.18% in Germany.

### 3.3. Direct Environmental Impact

**Figure 3:**
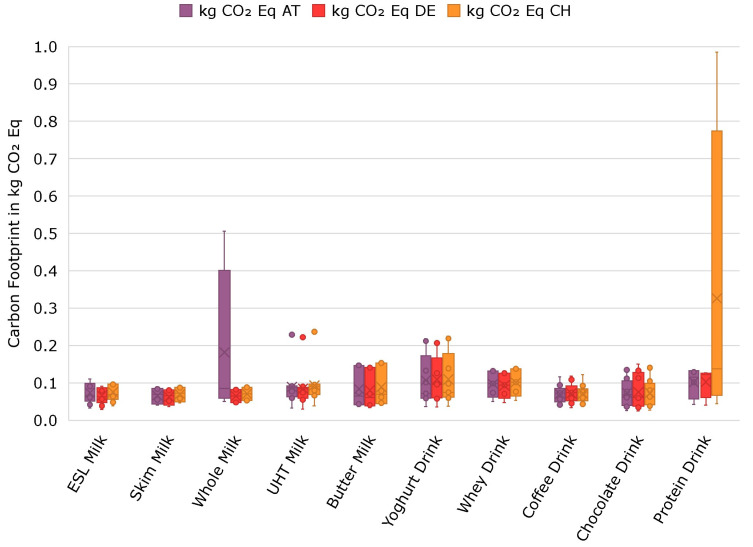
Carbon footprint of dairy product packaging in five product groups for the countries Austria (AT), Germany (DE) and Switzerland (CH).

A notable difference was observed in the carbon footprint of liquid dairy product packaging (Figure 3). The weight and materials used are of pivotal importance in determining the footprint. The variations observed between the three countries can be attributed primarily to differences in transportation distances and the composition of the electrical grid. A closer examination of the UHT milk category reveals outliers with a CO_2_ eq of approximately 0.2 kg. These emissions stem from the use of an HDPE bottle, resulting in approximately three times higher emissions compared to beverage cartons. One HDPE bottle containing UHT milk exhibited 0.222 kg CO_2_ eq. in total in Germany, of which 0.126 is attributed to the manufacturing of the packaging, 0.00162 kg CO_2_ eq. to the transport, 0.0146 kg CO_2_ eq. to the distribution and 0.0797 kg CO_2_ eq. to the end-of-life treatment. Comparably, the emissions of a beverage carton (sample number T42) are 0.082 kg CO_2_ eq. in total, with 0.061 kg CO_2_ eq. for manufacturing, 0.00119 kg CO_2_ eq. for transportation, 0.0108 kg CO_2_ eq. for distribution and 0.00905 kg CO_2_ eq. for end-of-life treatment.

Further, the results demonstrate that coffee drinks exhibit the most pronounced differences in emissions across different packaging types. In Austria, Germany and Switzerland, the emissions associated with a fiber-based can are seven times those of an aluminum can and 3.5 times higher than those of a PET or HDPE bottle, with respective values of 0.356, 0.315 and 0.450 kg CO_2_ eq.

### 3.4. Use of Certified Renewable Resources and Recyclate

It is a common practice to utilize renewable raw materials in the production of packaging for milk, particularly in the case of composite beverage cartons. A total of 46 samples were found to contain renewable resources, with some cartons achieving up to 97.46% renewable content through the use of biologically based plastics, such as BIO-PE for the LDPE layer and screw cap. In addition to the beverage cartons, a single fiber-based can was evaluated and exhibited a renewable material content of 76.78%. The HDPE bottle was accompanied by a label made of paper, which indicated a renewable material content of 9.22%. The range of renewable content in beverage cartons across the assessed milk categories was found to vary considerably, from 60.55% to 97.46%. The mean value for ESL and fresh milk was found to be higher, at 82.38%, compared to the content of renewable materials used in packaging for UHT milk, which was found to be 73.78%. This difference can be attributed to the inclusion of additional aluminum layers in UHT milk packaging, which affects the overall ratio of the material composition. All beverage cartons were constructed from certified materials.

In mixed milk drinks, renewable raw materials were predominantly confined to composite beverage cartons and fiber-based cans, with the highest proportion observed in a beverage carton (91.57%), in addition to one to-go cup with a fiber-based label. A mere six samples were found to utilize certified materials, all of which were beverage cartons. Of the 17 beverage cartons, 13 were composed of certified materials, while 4 were made from fibers sourced from uncertified sources. The fiber-based can was composed of certified fibers.

In the milk product category, the use of recyclate was limited to two PET bottles, with values of 40.21% and 86.68%. The packaging with the highest proportion of recycled material was a 100% rPET bottle with an HDPE cap, which was manufactured using virgin material in accordance with the specifications, resulting in a recyclate content of 86.68% in the overall packaging.

In the category of yoghurt drinks, three PET bottles contained recycled material to the extent of 39.99%, 39.40% and 74.62%. One PET bottle for coffee drinks was found to contain a similar content of recycled material, at 39.39%.

### 3.5. Packaging Efficiency

In the category of milk products, an LDPE pouch containing skim milk was observed to demonstrate the most efficient packaging, with a value of 1.56%. A PET bottle demonstrated a packaging efficiency of 2.85%, and beverage cartons of different designs varied between 2.58 and 3.09%. For UHT milk, an HDPE bottle showed a packaging efficiency of 3.63%.

In the non-fermented mixed milk drinks product category, most of the analysis focused on smaller containers (230 mL to 500 mL). It is often observed that packaging efficiency is negatively impacted by smaller portion sizes. The to-go cups, which consist of several components, exhibited values between 3.36 and 4.57% in this category. The best result was achieved by an aseptic composite beverage carton for drinking cocoa with a value of 2.99%. The highest values were achieved by an HDPE and a PET bottle with 7.28 and 7.51%, respectively.

For both fermented and non-fermented mixed milk drinks, the results for PET bottles show high variations, with values between 3.46 to 7.51%, and HDPE bottles expressed packaging efficiencies of 4.82 to 7.43%. The least effective result was observed for the smallest packaging, the LDPE pouch, with a value of 7.85%. The total weight of the packaging increases due to the inclusion of the HDPE spout, which weighs almost as much as the pouch itself. Furthermore, the filling weight is only 70 g.

### 3.6. Consumer Involvement

It is not a requirement to separate the components of beverage cartons in order to facilitate their recycling. In Austria and Germany, dedicated waste streams have been established to ensure the separate recycling of specific materials. Therefore, the rating category 3 was applied to all beverage cartons. The same rating was applied to PET and HDPE bottles, where material combinations permitted separation during the recycling process and the recyclability of all materials. This leads to the conclusion that the product categories for milk are fully evaluated with the rating category 3. With regard to fermented mixed milk drinks, one PET bottle was assigned a rating of category 1, given that separation of the PS sleeve from the PET bottle for enhanced recycling is essential and additionally indicated on the label for the consumer. In the case of other PET and HDPE bottles (*n* = 5), a rating of 2 was assigned, as the requisite indication for the consumer was absent. Regarding non-fermented drinks, three PET bottles and two to-go cups were assigned a rating of category 1, while four PET bottles and three to-go cups were assigned a rating of category 2. Beverage cartons, fiber-based and aluminum cans, as well as two PET bottles, were assigned a rating of category 3.

## 4. Discussion

The assessment of the sustainability of packaging is a complex process, as it requires consideration of a multitude of criteria. This study analyzed the packaging for milk and mixed milk products on the German-speaking market for the product categories of ESL milk, fresh milk, UHT milk, buttermilk, yoghurt drinks, whey drinks, coffee drinks, cacao and protein drinks. In addition, key sustainability indicators were collected. The following important findings were obtained:

1. A significant proportion of packaging on the market in Germany and Austria is below the minimum recyclability requirement of 70% set out in the current draft of the PPWR [14]. In Switzerland, the recyclability is even lower; this is due to the low number of established material collection streams and consequently high rates of incineration;
2. Important factors determining recyclability are non-compatible material combinations and packaging design, in particular the choice of fully sleeved packaging solutions that can reduce sortability by NIR;
3. There are significant differences in the packaging efficiency of the packaging formats on the market. Some packaging solutions are considerably heavier than their counterparts, resulting in the unnecessary consumption of resources. Furthermore, the carbon footprint of packaging materials reveals differences of up to five times for certain product categories. This discrepancy in packaging efficiency has a detrimental impact on the environment.

### 4.1. Comparison of Recyclability and Carbon Footprint

When selecting sustainable packaging options, there can be a conflict between the principles of recyclability and the reduction of carbon footprints.

**Figure 4:**
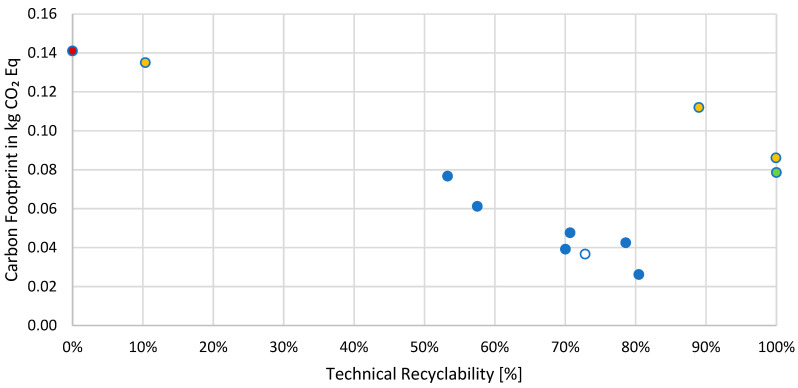
Comparison of the results of the streamlined LCA and recyclability analysis for the product category of chocolate drinks in Austria. Depicted are different packaging types in red (HDPE bottle), yellow (PET bottle), blue (beverage carton), white (fiber-based can) and green (aluminum can).

From Figure 4, it can be seen that fiber-based beverage cartons showed the lowest global warming potential (GWP), together with a fiber-based carton/can. The recyclability of beverage cartons depends on the composition (mainly the plastic content) and lies between 53.30% and 80.47%. The aluminum can (green) performed well in the comparative analysis, giving slightly higher GWP emissions than most of the beverage cartons but showing a recyclability of 100%. PET bottles are mainly well recyclable, with values between 99.95% and 89.00%, but the GWP for PET bottles is higher than for beverage cartons and the aluminum can. One PET bottle was non-recyclable due to a full LDPE sleeve, resulting in incorrect material assignment in the sorting plant. The final packaging type is represented by bottles made of HDPE, marked in red, which exhibited the lowest recyclability (0.00%) and a high carbon footprint (0.14 kg CO_2_ eq). The low recyclability of the packaging format assessed was due to a sleeve preventing the NIR detection of the can in an automatic sorting process, leading to an allocation in the wrong material stream. This shows that simple Design for Recycling recommendations are not always followed, leading to non-sustainable packaging solutions on the market.

High variability was detected between packaging formats of a given type, such as beverage cartons (0.03 to 0.08 kg CO_2_ eq), and between different packaging categories. Differences between the GWP emissions of the packaging format with the lowest value (0.03 kg CO_2_ equivalent) and the packaging format with the highest value (0.14 kg CO_2_ eq) are significant and show potential for improvement for many packaging formats on the market.

In conclusion, the results for chocolate drink packaging demonstrate the necessity for the further optimization of packaging to achieve the goals and objectives set by EU regulations in regard to recyclability and environmental effects. This includes adapting material combinations to ensure adequate separation in the recycling process, providing information for consumers and incorporating perforations to enhance consumer engagement, as well as implementing pre-sorting at the consumer level.

The results for recyclability and carbon footprint for other product categories such as coffee drinks lead to similar conclusions, as the same packaging types are prevalent. For product categories with a higher share of beverage cartons, the results are less diverse and show accumulations of recyclability between 60 and 80% and a similar carbon footprint.

In the assessment of the carbon footprint, the quality of product protection is not considered and could be topic for further research.

### 4.2. Material Usage

The utilization of post-consumer recyclate in the food sector is constrained by legal requirements [39], as the conversion of food packaging waste into new food packaging material presents a number of challenges, including safety issues. The presence of unintentionally added substances can reach relatively higher levels in food packaging made from recycled materials [40] Therefore, the use of recycled materials from non-food plastics is prohibited in the production of fresh food packaging, in accordance with the regulations set by EU Regulation 10/2011 and EFSA standards. This is because recycled materials fail to fulfill the essential criteria for odor or contamination control [41] With regard to plastics, the sole permitted use in the food sector is that of rPET for bottles.

This regulation also has an impact on this assessment. As can be drawn from the results, very few samples contain recyclate. These were, with few exceptions, only found in PET bottles. Another aspect is the use of renewable materials in order to substitute plastics and, in the case of using renewable resources, to resort to material from certified sources.

### 4.3. Implications for Future Legislation

The assessment of the sustainability of liquid dairy product packaging and its results are highly relevant for upcoming regulations, indicating current potential for improvement for the packaging industry.

The recyclability of packaging is a central point of the Packaging and Packing Waste Regulation, stating in Article 6 that all packaging should be recyclable [14]. In order to archive optimal recyclability, several factors in the design of packaging need to be considered. For one, packaging made of diverse materials must be separable so materials can enter the correct waste stream. This means that materials need be detectable by NIR identification; therefore, sleeves covering a large quantity of the packaging surface should only be used when compatible with the sorting process, mainly NIR detection. Additionally, printing inks, as well as black-colored plastics, have been proven to inhibit recyclate quality [4].

Article 7 then states clear minimum limit values for recycled content in plastic packaging. In the currently available version, the limit value for contact-sensitive packaging made from PET is set to be 30% from 2030 onwards [14]. This limit was only met by 4 out of the 15 sampled PET bottles. The limit for contact-sensitive packaging made from plastics other than PET is set at 10%, which is also not achieved by the majority of available dairy packaging options currently on the market.

With regard to the parameter of packaging efficiency, Article 9 of the PPWR states vague limitations to the extent of packaging weight and volume [14] It states that “packaging shall be designed so that its weight and volume is reduced to the minimum necessary for ensuring its functionality taking account of the material that the packaging is made of”. As packaging minimization has to happen on the account of the different packaging materials, no direct switch from glass to plastic packaging is needed in order to ensure a higher packaging efficiency. This Article includes the argument that double walls, false bottoms and unnecessary layers have to be eliminated. Those characteristics could not be identified in dairy packaging. The results of this study allow recommendations to be deduced, with one such recommendation relating to the filling volume and the observation that optimized portion sizing enhances packaging efficiency. Furthermore, this study demonstrates that a simplified design, as exemplified by to-go cups, can also result in a reduction in material usage. However, significant variations in material usage were identified for PET and HDPE bottles, ranging from 3.46% to 7.51% and from 4.82% to 7.43%, respectively. This calls into question the efficacy of simplified designs in these contexts. Beverage cartons, which share similarities in design and material composition, exhibit a minimal variation in packaging efficiency, as evidenced by the results for skim milk, which range from 2.58% to 3.09%.

## 5. Conclusions

This comprehensive study provides a robust foundation for evaluating the current sustainability of packaging for liquid dairy products on the market in German-speaking countries. From the aforementioned findings, recommendations for action can be derived to ensure that the dairy and packaging industry is able to fulfill the requirements of the PPWR in the future. A key objective of the PPWR is to achieve a minimum of 70% recyclability [14]. This target has, thus far, only been met by a limited number of packaging samples, highlighting a significant need for action regarding material composition and combination, as well as the usage of colorants. Furthermore, there is a pressing requirement to enhance the efficiency and reduce the environmental impact of packaging. The results for these two aspects exhibit considerable discrepancies.

The objective of the future optimization of packaging for dairy products needs to be to minimize the consumption of resources and the negative environmental impact of the packaging, while simultaneously improving the usage of recycled and renewable materials. This necessitates a multi-dimensional optimization, which should not be limited to a single criterion such as recyclability, regardless of its importance.

In the forthcoming years, the packaging industry and brand manufacturers will be compelled to accord greater priority to sustainability in the development of new packaging solutions or the modification of existing packaging, while ensuring product protection. As the EU tightens its regulations on packaging sustainability, it will become imperative for brand manufacturers and the packaging industry to select solutions that not only comply with legal requirements but also meet consumer expectations, reduce environmental impacts and align with the principles of a circular economy. Those that proactively invest in sustainable packaging innovation are likely to benefit from enhanced market competitiveness and long-term success.

## Figures and Tables

**Table 1:** Types of packaging assessed in the different product categories.

Product Category	PET Bottle	HDPE Bottle	PS Bottle	Beverage Carton	Pouch	To-Go Cup	Can (Aluminum)	Can (Fiber)
ESL Milk	2			7				
Skim Milk				3	1			
Whole Milk				4				
UHT Milk		1		13				
Butter Milk		1	1	4				
Yoghurt Drink	3	2		3	1			
Whey Drink	2			3				
Coffee Drink	2					5	1	1
Cacao	3	1		6			1	1
Whey Drink	3			1				

**Table 2:** Results of the market screening for liquid dairy products in Austria.

Category	Subcategory	Packaging Type	Number of Available Products on the Market	Market Share in %
Milk	Fresh and ESL Milk	Reuseable Glass Bottle	8	15
		Beverage Carton	41	79
		Plastic Bottle	3	6
	UHT Milk	Beverage Carton	15	100
Mixed Milk Products	Buttermilk	Beverage Carton	23	96
		Plastic Bottle	1	4
	Yoghurt Drink	Cup	2	6
		Beverage Carton	3	9
		Plastic Bottle	29	85
	Whey Drink	Beverage Carton	19	86
		Plastic Bottle	3	14
	Coffee Drink	Aluminum Can	6	11
		Cup	37	67
		Reusable Glass Bottle	1	2
		Beverage Carton	3	5
		Plastic Bottle	3	5
		Fiber-Based Can	5	9
	Chocolate Drink, Vanilla Drink, Cacao	Cup	6	8
		Reusable Glass Bottle	2	3
		Beverage Carton with Straw	9	13
		Beverage Carton	17	24
		Plastic Bottle	32	45
		Fiber-Based Can	5	7

## Data Availability

The original contributions presented in this study are included in the article/Supplementary Material. Further inquiries can be directed to the corresponding author.

## Supplementary Materials

The following supporting information can be downloaded at: www.mdpi.com/article/10.3390/foods14020195/s1, Supporting information: Assessment Data.
